# Exploring factors associated with autism spectrum disorder diagnosis among children in primary care clinics in Kelantan, Malaysia

**DOI:** 10.1186/s12875-026-03424-0

**Published:** 2026-06-17

**Authors:** Farah Munirah Mior Mazlan, Raishan Shafini Bakar, Suhaily Mohd Hairon, Masliza Yusoff, Nik Normanieza Nik Man

**Affiliations:** 1 Department of Community Medicine, School of Medical Sciences, Universiti Sains Malaysia, Health Campus, Kubang Kerian, Kota Bharu, Kelantan 16150 Malaysia; 2 Kota Bharu Health Clinic, Kota Bharu District Health Office, Kota Bharu, Kelantan Malaysia; 3 Maternal and Child Health Unit, Kelantan State Health Department, Kota Bharu, Kelantan Malaysia

**Keywords:** Autism spectrum disorder, Associated factors, Primary healthcare, Sociodemographic factors, Birth order, Kelantan

## Abstract

**Background:**

Autism spectrum disorder (ASD) is a neurodevelopmental condition characterised by impairments in social communication and restricted or repetitive behaviors. In Malaysia, timely identification within routine primary care services remains challenging, particularly outside major urban centres. This study examined factors associated with ASD diagnosis among children attending government primary care clinics in Kelantan to inform strategies for strengthening primary care developmental surveillance.

**Methods:**

A clinic-based cross-sectional study was conducted from March to September 2025 in five districts. Using purposive sampling, 207 children aged 18 months to 12 years were recruited during routine clinic visits, developmental presentations and primary care visits. Sociodemographic and clinical data were captured via a structured proforma. All enrolled children underwent Sensory-Behavior Profile screening followed by clinical assessment utilizing the Diagnostic and Statistical Manual of Mental Disorders, Fifth Edition criteria regardless of screening result. Simple and multiple logistic regression were applied to examine associations between child and parental characteristics and ASD diagnosis.

**Results:**

Of the 207 children analysed, 29 were newly diagnosed with ASD during the study assessment and 178 were classified as non-ASD. In the multivariable analysis, two factors remained independently associated with ASD diagnosis. Older age at assessment was associated with higher odds of ASD diagnosis (aOR 1.30, 95% CI 1.10,1.55; *p* = 0.003), a finding that likely reflects later recognition, presentation, referral, or diagnostic confirmation within primary care pathways rather than an etiologic risk factor. First-born children were more likely to have an ASD diagnosis than second- or later-born children (aOR 2.48, 95% CI 1.08,5.71; *p* = 0.033).

**Conclusions:**

These findings support the strengthening of primary care developmental surveillance through consistent routine screening, improved clinician competency in developmental surveillance, increased parent awareness, and clear, time-bound referral pathways linked to timely assessment capacity within routine child health services.

## Introduction

Autism spectrum disorder (ASD) is a long-term neurodevelopmental condition characterised by differences in social communication and interaction, restricted or repetitive behaviors, and atypical sensory responses [1–3]. Although early features may be detectable in the first two years of life, the clinical presentation is heterogeneous and can evolve with age, contributing to wide variation in the timing of recognition and diagnostic confirmation [1, 4, 5]. ASD is also commonly accompanied by co-occurring conditions, including language delay, intellectual disability, attention-deficit/hyperactivity disorder, anxiety, sleep disturbances, epilepsy, and gastrointestinal symptoms, which can complicate case recognition and increase caregiving demands and service utilisation [5, 6]. Timely identification matters because evidence-based developmental interventions initiated in the preschool years are associated with improvements in language, social communication, and adaptive functioning [7]. However, many children are still diagnosed later than recommended, with reports of substantial delays between first parental concerns and diagnosis, which can reduce opportunities for time-sensitive intervention and prolong family stress [8]. The economic implications are also considerable, extending beyond healthcare costs to specialised education, caregiver time, and productivity losses [9, 10].

In most health systems, primary care is the main entry point for routine child health care and developmental surveillance. Accordingly, primary care clinicians are positioned to elicit caregiver concerns, observe developmental progress during child visits, apply screening tools, provide anticipatory guidance, and coordinate referrals for confirmatory assessment and early intervention. Evidence from routine primary care practice shows that wide screening uptake does not necessarily lead to prompt specialist assessment or diagnosis; low referral rates and poor follow-up contribute to diagnostic delays [8]. Clinician- and system-level barriers can further weaken early identification, including limited consultation time, low familiarity with screening instruments, insufficient training and confidence, and uncertainty about local referral resources [11, 12]. These challenges are especially important when ASDs exhibit sensory-behavioral features, which may require additional time and clinical experience in primary care encounters [12]. In this context, culturally appropriate, feasible screening approaches and the capacity to act on screening results through efficient referral pathways are central to strengthening early identification in primary care [8, 13].

Across settings, reported ASD prevalence has increased over recent decades, likely reflecting improved awareness, broadened diagnostic concepts, and more systematic identification. The World Health Organisation estimates that approximately one in 100 children are identified with ASD, whereas surveillance in the United States has reported higher prevalence [14, 15]. In Malaysia, reliable population-level estimates remain limited, and the absence of a national registry has been noted as a constraint for accurately characterising the burden and planning services [16, 17]. Primary care clinics are the principal platform for child developmental surveillance in Malaysia, but progression from early concerns to diagnostic confirmation can be constrained by variable autism literacy, differences in parental help-seeking, and uneven access to specialist assessment and early intervention services, particularly outside major urban centres [18–21]. Kelantan is a predominantly rural northeastern state of Peninsular Malaysia where government health clinics provide most routine maternal and child health services, making primary care pathways a critical locus for strengthening early identification and equitable access to assessment.

Epidemiological research has examined the associations between ASD diagnosis and child and parental characteristics such as sex, parental age, maternal comorbidities, educational attainment, birth outcomes, and birth order [5, 22, 23]. Nevertheless, much of the existing evidence originates from high-income settings, and observed associations may not translate directly to low- and middle-income contexts where health service organisation, developmental surveillance practices, and referral pathways differ [24–27]. In primary care settings, some observed characteristics may reflect how diagnoses are identified such as differences in recognition, care-seeking patterns, and access to confirmatory assessment rather than representing causal exposures.

Local evidence on factors associated with ASD diagnosis within Kelantan primary care remains limited. Understanding diagnostic associations in routine clinic populations can inform targeted developmental surveillance, support timely referral, and guide service planning across districts. Therefore, this study aimed to explore factors associated with ASD diagnosis among children attending government primary care clinics in Kelantan, Malaysia, addressing an important evidence gap relevant to strengthening primary care practice and public health planning.

## Materials and methods

### Study design, participants, sample size, and sampling method

A clinic-based cross-sectional study was conducted between March and September 2025 in government primary care clinics in Kelantan, a northeastern state in Peninsular Malaysia comprising 10 districts: Kota Bharu, Bachok, Pasir Puteh, Pasir Mas, Tumpat, Tanah Merah, Machang, Kuala Krai, Jeli, and Gua Musang. This study was part of a broader project evaluating the newly developed Sensory-Behavior Profile (S-BP) screening tool and aimed to identify factors associated with ASD diagnosis among children attending primary care services.

Data collection was carried out in clinics located within five selected districts. Study sites were selected purposively in consultation with the Kelantan State Health Department to ensure feasibility within routine primary care settings. Type 1 to Type 4 clinics with an average daily attendance exceeding 150 patients were prioritised, as these facilities were expected to provide an adequate pool of eligible participants during the study period. This approach also helped minimise disruption to routine service delivery while maintaining the study’s relevance to real-world primary care practice.

**Fig. 1:**
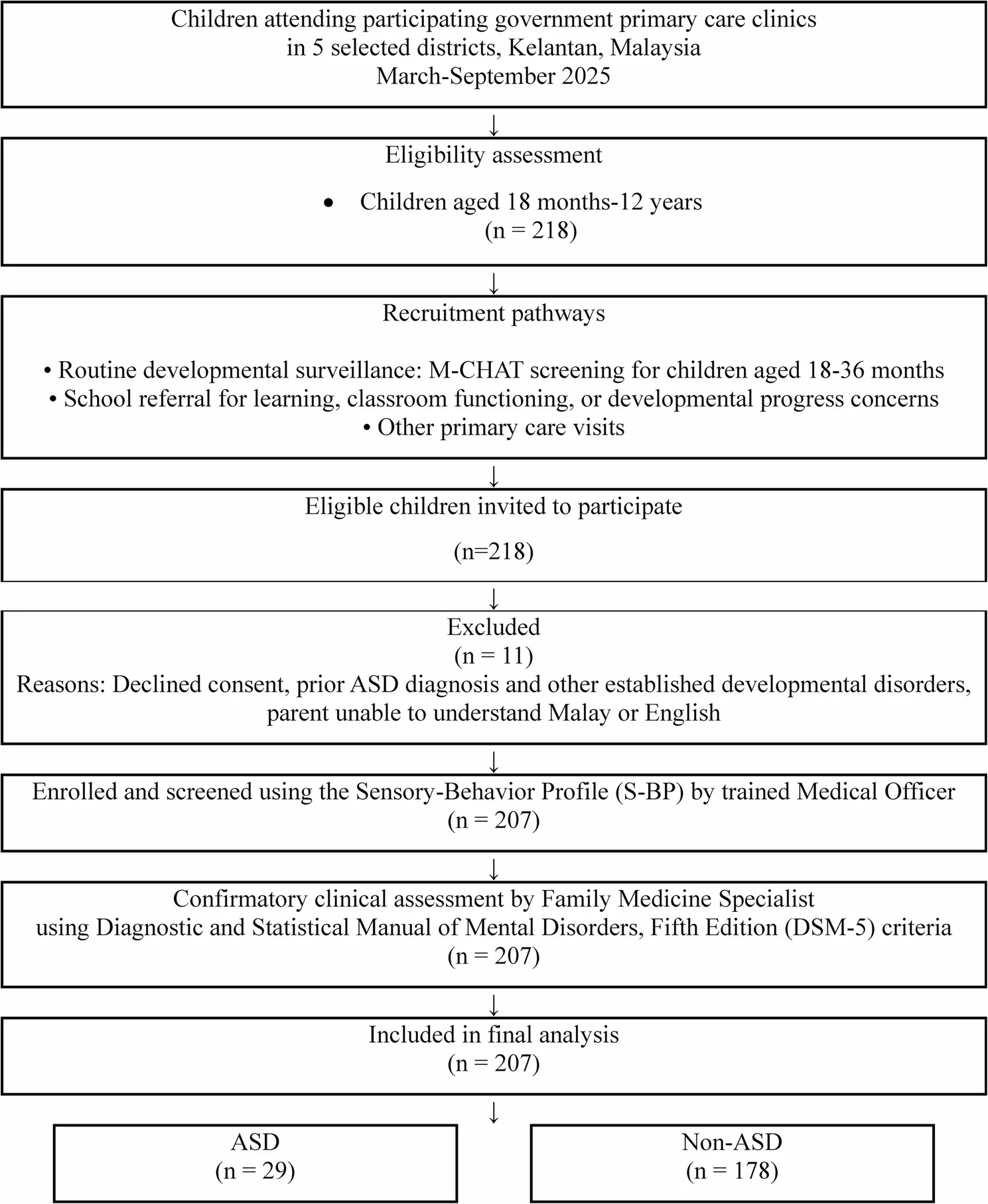
Flow of study recruitment

Eligible participants were children aged 18 months to 12 years who attended the selected clinics for developmental-related services or other primary care encounters. Recruitment pathways comprised routine developmental surveillance with M-CHAT screening for children aged 18 to 36 months; school referral for concerns about learning difficulties, classroom functioning, or developmental progress; and identification during other primary care visits. Children with a previous diagnosis of ASD or other established developmental disorders were excluded to ensure that the ASD outcome represented newly identified cases detected through the study assessment pathway. Children were also excluded if their parents declined consent or were unable to understand Malay or English. Parents who could understand Malay or English were invited to participate, and written informed consent was obtained before enrolment. The flow of study recruitment is shown in Fig. 1.

The sample size was calculated using PS software for dichotomous outcomes, based on the proportion in the unexposed group (P0 = 0.20) and the estimated proportion in the exposed group (P1 = 0.40) derived from previous studies [28]. The calculation was performed with power set at 0.8 and α = 0.05. After allowing for an anticipated 20% dropout rate, the minimum required sample size was 203 participants. As this study was conducted alongside the psychometric evaluation of the S-BP, which required a larger sample size, the final sample size was set at 207 participants.

### Screening and diagnostic assessment

All participating children were screened using the Sensory-Behavior Profile (S-BP), a locally developed screening instrument created by a multidisciplinary team at the Kelantan Autism Care Centre. The S-BP comprises 34 items across two domains: Sensory and Behavior. For the Sensory domain, scores of 15–32 are considered typical, whereas scores outside this range suggest sensory hyperresponsiveness or hyporesponsiveness. For the Behavior domain, scores of 20–47 are considered typical, with scores outside this range indicating behavioral patterns that may warrant further evaluation.

The S-BP was evaluated as part of the broader study and was used to support screening in the present analysis. The psychometric evaluation demonstrated favourable findings, with item-level content validity indices ranging from 0.60 to 1.00 and a scale-level content validity index exceeding 0.90. The item with an I-CVI of 0.60 was retained after expert review because it was considered clinically relevant to sensory-behavioral presentation and was subsequently refined for wording clarity and local interpretability. Face validity was high, with item-level face validity indices ranging from 0.92 to 1.00 and a scale-level face validity index of 0.95. Inter-rater reliability was substantial (κ = 0.88; 95% CI 0.83,0.91). In the concurrent diagnostic accuracy analysis, the S-BP showed a sensitivity of 83%, specificity of 89%, positive predictive value of 55%, and negative predictive value of 97%.

In this study, the S-BP was positioned as a supplementary screening instrument within existing developmental surveillance processes in Malaysian primary care. It was designed to support medical officers’ clinical workflow by providing a structured approach to assessing sensory-behavioral features that may indicate the need for further assessment. This complements current child health services, where tools such as M-CHAT are commonly used by public health nurses for initial toddler screening, followed by medical officer review and referral for confirmatory assessment when developmental concerns are identified [2, 29]. For the purposes of this study, the S-BP was administered to all eligible children as a screening instrument only and was not used to establish the final ASD diagnosis.

Following S-BP screening, all enrolled children, regardless of screening result, underwent confirmatory clinical assessment by a Family Medicine Specialist. This assessment was conducted by 15 Family Medicine Specialists working in participating government primary care clinics as part of the established primary care referral pathway. ASD status was classified as ASD or non-ASD based on DSM-5 criteria and was used as the reference standard for the diagnostic accuracy analysis. The assessment included clinical and developmental history, caregiver-reported concerns, behavioral observation, and review of relevant clinic information. No additional standardised diagnostic instrument, such as the Autism Diagnostic Observation Schedule or Autism Diagnostic Interview-Revised, was used in this study.

To reduce assessment bias, the assessing Family Medicine Specialist was blinded to the S-BP screening result during the clinical evaluation. Formal inter-rater reliability testing between assessors was not performed. However, the involvement of multiple Family Medicine Specialists reflects real-world primary care practice across the participating districts.

### Assessment tool

A structured proforma was used to obtain detailed sociodemographic and clinical information from parents to capture factors potentially associated with ASD diagnosis. The tool captured child-related variables such as age, sex, birth weight, gestational age at delivery, mode of delivery, number of siblings, birth order (first-born versus second- or later-born), the presence of siblings diagnosed with ASD, ethnicity, immunisation status, and existing comorbidities. The parental information included maternal and paternal age at the child’s birth, maternal history of gestational diabetes mellitus or hypertension during pregnancy, parental educational attainment, and monthly household income. All the information recorded in the proforma was subsequently entered into Microsoft Excel and exported to IBM SPSS version 29 for statistical analysis.

### Data collection

Data collection was conducted during clinic sessions at the participating primary care clinics. Two trained medical officers identified eligible children, explained the study procedures and the voluntary nature of participation to parents, obtained written informed consent, and administered the S-BP before referral to a Family Medicine Specialist for confirmatory assessment. Parents were then interviewed using the structured proforma. Whenever possible, the information provided was cross-checked against clinic medical records to improve data accuracy and completeness. The final ASD classification assigned by the Family Medicine Specialist was recorded as the diagnostic outcome for analysis.

### Statistical analysis

All data were analysed using IBM SPSS version 29. Descriptive statistics were used to summarise all study variables. Simple logistic regression was first performed to examine the univariable associations between individual child and parental factors and ASD diagnosis.

Variables with a *p* < 0.25 in the univariable analysis were considered for multivariable modelling to minimise premature exclusion of potentially important covariates. Given the modest number of ASD cases, candidate variables were further reviewed based on clinical plausibility, relevance to ASD diagnostic pathways, and model stability. A parsimonious multiple logistic regression model was then developed to estimate adjusted associations with ASD diagnosis.

Model diagnostics and assumptions were assessed, including multicollinearity, interaction terms, goodness-of-fit using the Hosmer-Lemeshow test, and discrimination using the area under the receiver operating characteristic curve. The area under the curve was reported to describe model discrimination. Complete-case analysis was performed, as there were no missing values for variables included in the final model. Statistical significance was set at *p* < 0.05.

## Results

### Sociodemographic characteristics of participants

A total of 207 children were included in the analysis. Of these, 29 children (14.0%) were newly diagnosed with ASD during the study assessment, while 178 children (86.0%) were classified as non-ASD. Table 1 summarises the sociodemographic characteristics of the study population, including child-related and parental-related factors, and compares these characteristics between children with and without ASD.

**Table 1:** Sociodemographic characteristics of the study population and comparison of between children with and without ASD

Variables	Mean (SD)	Overall (*n* = 207)	ASD (*n* = 29)	No ASD (*n* = 178)
Child factors				
Child Age (years)	7.38 (3.35)			
Child Age				
< 7 years old 7–9 years old 10–12 years old		64 (30.9) 66 (31.9) 77 (37.2)	0 (0.0) 17 (58.6) 12 (41.4)	64 (36.0) 49 (27.5) 65 (36.5)
Sex				
Male Female		132 (63.8) 75 (36.2)	18 (62.1) 11 (37.9)	114 (64.0) 64 (36.0)
Birth weight (g)				
< 1500 1500–2499 * ≥* 2500		3 (1.4) 15 (7.2) 189 (91.3)	1 (3.4) 3 (10.3) 25 (86.2)	2 (1.1) 12 (6.7) 164 (92.1)
Gestational age				
< 37 weeks * ≥* 37 weeks		17 (8.2) 190 (91.8)	5 (17.2) 24 (82.8)	12 (6.7) 166 (93.3)
Types of delivery				
Normal Instrumental delivery Caesarean section		171 (82.6) 2 (1.0) 34 (16.4)	22 (75.9) 0 (0.0) 7 (24.1)	149 (83.7) 2 (1.1) 27 (15.2)
Birth order				
First-born Second-or later-born		72 (34.8) 135 (65.2)	14 (48.3) 15 (51.7)	58 (32.6) 120 (67.4)
Sibling with ASD				
Yes No		13 (6.3) 194 (93.7)	1 (3.4) 28 (96.6)	12 (6.7) 166 (93.3)
Ethnicity				
Malay Chinese Others		200 (96.6) 5 (2.4) 2 (1.0)	29 (100.0) 0 (0.0) 0 (0.0)	171 (96.1) 5 (2.8) 2 (1.1)
Immunisation status				
Complete Not complete		201 (97.1) 6 (2.9)	28 (96.6) 1 (3.4)	173 (97.2) 5 (2.8)
Having comorbidities				
Yes No		13 (6.3) 194 (93.7)	1 (3.4) 28 (96.6)	12 (6.7) 166 (93.3)
Parental factors				
Maternal age at child’s birth				
< 20 20–39 * ≥* 40		10 (4.8) 180 (87.0) 17 (8.2)	4 (13.8) 22 (75.9) 3 (10.3)	6 (3.4) 158 (88.8) 14 (7.9)
History of gestational diabetes				
Yes No		43 (20.8) 164 (79.2)	7 (24.1) 22 (75.9)	36 (20.2) 142 (79.8)
History of hypertension in pregnancy				
Yes No		18 (8.7) 189 (91.3)	1 (3.4) 28 (96.6)	17 (9.6) 161 (90.4)
Maternal educational level				
No formal education Primary Secondary Tertiary		5 (2.4) 23 (11.1) 119 (57.5) 60 (29.0)	1 (3.4) 5 (17.2) 16 (55.2) 7 (24.1)	4 (2.2) 18 (10.1) 103 (57.9) 53 (29.8)
Paternal age at child’s birth				
< 20 20–39 * ≥* 40		4 (1.9) 164 (79.2) 39 (18.8)	0 (0.0) 24 (82.8) 5 (17.2)	4 (2.2) 140 (78.7) 34 (19.1)
Paternal educational level				
No formal education Primary Secondary Tertiary		20 (9.7) 23 (11.1) 114 (55.1) 50 (24.2)	0 (0.0) 7 (24.1) 16 (55.2) 6 (20.7)	20 (11.2) 16 (9.0) 98 (55.1) 44 (24.7)
Household monthly income (RM)				
< 1000 1000–2999 3000–4999 5000–6999 * ≥* 7000		61 (29.5) 93 (44.9) 32 (15.5) 12 (5.8) 9 (4.3)	5 (17.2) 16 (55.2) 6 (20.7) 2 (6.9) 0 (0.0)	56 (31.5) 77 (43.3) 26 (14.6) 10 (5.6) 9 (5.1)

### Child-related factors

The mean age of the participating children was 7.38 years (SD = 3.35). Overall, 30.9% were aged below 7 years, 31.9% were aged 7–9 years, and 37.2% were aged 10–12 years. Children in the ASD group were generally older than those in the non-ASD group. None of the children diagnosed with ASD were aged below 7 years, while 58.6% were aged 7–9 years and 41.4% were aged 10–12 years. In comparison, among children without ASD, 36.0% were aged below 7 years, 27.5% were aged 7–9 years, and 36.5% were aged 10–12 years.

The study sample comprised more boys than girls. Among children diagnosed with ASD, 62.1% were boys and 37.9% were girls, giving a male-to-female ratio of approximately 1.6:1. A broadly similar sex distribution was observed in the non-ASD group, where 64.0% were boys and 36.0% were girls.

Most children in both groups had a birth weight above 2,500 g. However, lower birth weight categories were slightly more frequent among children with ASD, with 10.3% having low birth weight and 3.4% having very low birth weight, compared with 6.7% and 1.1%, respectively, among children without ASD. Preterm birth (< 37 weeks) was also more frequent among children with ASD, occurring in 17.2% of the ASD group compared with 6.7% of the non-ASD group.

Normal vaginal delivery was the most common mode of delivery in both groups. Among children with ASD, 75.9% were delivered by normal vaginal delivery and 24.1% by caesarean section, with no instrumental deliveries reported. Among children without ASD, 83.7% were delivered by normal vaginal delivery, 15.2% by caesarean section, and 1.1% by instrumental delivery.

In relation to birth order, first-born children were more common in the ASD group than in the non-ASD group. Among children with ASD, 48.3% were first-born and 51.7% were second- or later-born. In contrast, among children without ASD, 32.6% were first-born and 67.4% were second- or later-born. A sibling history of ASD was uncommon in both groups, reported in 3.4% of children with ASD and 6.7% of children without ASD.

The study population was predominantly Malay. All children diagnosed with ASD were Malay, while the non-ASD group consisted mainly of Malay children, with small proportions of Chinese children and children from other ethnic groups. Completion of the routine immunisation schedule was high in both groups, involving 96.6% of children with ASD and 97.2% of children without ASD. Documented comorbidities were uncommon, reported in 3.4% of children with ASD and 6.7% of children without ASD.

### Parental-related factors

Most mothers were aged 20–39 years at the time of the child’s birth. This age group accounted for 75.9% of mothers in the ASD group and 88.8% in the non-ASD group. A higher proportion of mothers of children with ASD were younger than 20 years at delivery compared with mothers of children without ASD. A history of gestational diabetes mellitus was reported in 24.1% of mothers of children with ASD and 20.2% of mothers of children without ASD. Hypertension during pregnancy was less frequently reported among mothers of children with ASD than among mothers of children without ASD.

Secondary education was the most common maternal educational level in both groups. Among mothers of children with ASD, 55.2% had secondary education, followed by tertiary education, primary education, and no formal education. A similar pattern was observed among mothers of children without ASD, with secondary and tertiary education forming the largest categories.

Most fathers were also aged 20–39 years at the time of the child’s birth, representing 82.8% of fathers in the ASD group and 78.7% in the non-ASD group. Paternal education showed a broadly similar pattern between groups, with secondary education being the most common level. No fathers in the ASD group had no formal education, while 11.2% of fathers in the non-ASD group had no formal education.

Household monthly income was concentrated mainly within the RM1,000-RM2,999 category in both groups. This income category accounted for 55.2% of families of children with ASD and 43.3% of families of children without ASD. No families in the ASD group reported household income above RM7,000, whereas 5.1% of families in the non-ASD group were in this income category.

### Factors associated with ASD diagnosis among children in Kelantan

#### Simple logistic regression

The associations between child and parental characteristics and ASD diagnosis among children in Kelantan were examined using simple logistic regression. The results are summarised in Table 2. Several variables demonstrated associations with ASD diagnosis at *p* < 0.25 and were considered for multivariable modelling. These included child age, gestational age, type of delivery, birth order, maternal age at child’s birth and maternal educational level.

**Table 2:** Factors associated with ASD diagnosis among children in Kelantan by simple logistic regression (*n* = 207)

Variables	Crude OR (95% CI)	Wald Statistic (df)	*p*-value
Child factors			
Child age (years)	1.27 (1.07,1.50)	7.83 (1)	0.005
Birth weight			
* ≥* 2500 g < 2500 g	1 1.87 (0.57,6.15)	6.47 (1)	0.300
Sex			
Female Male	1 0.92 (0.41,2.07)	0.04 (1)	0.837
Gestational age			
* ≥* 37 weeks < 37 weeks	1 2.88 (0.93,8.90)	3.38 (1)	0.066
Types of delivery			
Caesarean section Normal/Instrumental	1 0.56 (0.22,1.44)	1.43 (1)	0.231
Birth order			
Second- or later-born First-born	1 1.93 (0.87,4.27)	2.65 (1)	0.104
Sibling with ASD			
No Yes	1 0.49 (0.06,3.95)	0.44 (1)	0.506
Immunisation status			
Complete Not complete	1 1.24 (0.14,10.97)	0.04 (1)	0.849
Having comorbidities			
No Yes	1 0.49 (0.06,3.95)	0.44 (1)	0.506
Parental factors			
Maternal age at child’s birth			
< 20 years old 20–39 years old * ≥* 40 years old	1 0.21 (0.06,0.80) 0.32 (0.05,1.90)	5.24 (1) 1.57 (1)	0.022 0.210
History of gestational diabetes			
No Yes	1 1.26 (0.50,3.17)	0.23 (1)	0.631
History of hypertension in pregnancy			
No Yes	1 0.34 (0.04,2.64)	1.07 (1)	0.302
Maternal educational level			
No formal education/Primary Secondary Tertiary	1 0.57 (0.20,1.62) 0.48 (0.15,1.61)	1.11 (1) 1.41 (1)	0.291 0.236
Paternal age at child’s birth			
< 40 * ≥* 40	1 0.88 (0.31,2.48)	0.06 (1)	0.812
Paternal educational level			
No formal education/Primary Secondary Tertiary	1 0.84 (0.32,2.21) 0.70 (0.22,2.27)	0.13 (1) 0.35 (1)	0.723 0.554
Household income			
< 3000 * ≥* 3000	1 1.13 (0.47,2.72)	0.07 (1)	0.792

### Multiple logistic regression

Variables meeting the initial screening criterion of *p* < 0.25 were reviewed for clinical plausibility and model stability before inclusion in the final parsimonious model. The final adjusted estimates are presented in Table 3. After adjustment, two variables remained significantly associated with ASD diagnosis: child age and birth order.

**Table 3:** Factors associated with ASD diagnosis among children in Kelantan analysed by multiple logistic regression (*n* = 207)

Variables	B	Adjusted OR (95% CI)	Wald Statistic (df)	*p*-value
Child factors				
Child age (years)	0.27	1.30 (1.10,1.55)	8.94 (1)	0.003
Birth order				
Second- or later-born		1		
First-born	0.91	2.48 (1.08,5.71)	4.54 (1)	0.033

Candidate variables were selected based on univariable screening at *p* < 0.25 and clinical plausibility

No multicollinearity or interaction

Hosmer-Lemeshow test, *p*-value = 0.099

Classification accuracy = 86.0%

Area under the ROC curve was = 70.1%

Older age at assessment was associated with higher odds of ASD diagnosis (aOR 1.30, 95% CI 1.10,1.55; *p* = 0.003). Given the clinic-based cross-sectional design, this association should be interpreted as reflecting later recognition, presentation, referral, or diagnostic confirmation within service pathways rather than an etiologic risk factor for ASD.

Birth order was also significantly associated with ASD diagnosis. First-born children had higher odds of ASD diagnosis than second- or later-born children (aOR 2.48, 95% CI 1.08,5.71; *p* = 0.033).

Model diagnostics revealed no evidence of multicollinearity or interaction, and the Hosmer-Lemeshow goodness-of-fit test was non-significant (*p* = 0.099), suggesting no evidence of poor model fit. The final model had a classification accuracy of 86.0%, and the area under the ROC curve was 0.701, indicating fair discrimination. These findings were interpreted as measures of model performance only and not as evidence of clinical prediction or screening utility.

## Discussion

This study examined factors associated with ASD diagnosis among children attending government primary care clinics in Kelantan and identified child age and birth order as the only variables independently associated with an ASD diagnosis in the multivariable analysis. These findings contribute to the limited Malaysian evidence base and are directly relevant to strengthening primary care developmental surveillance and referral pathways.

In this clinic-based cross-sectional study, the association between older age at assessment and ASD diagnosis should not be interpreted as evidence that age is an etiologic risk factor for ASD. Instead, it more likely reflects later recognition, delayed presentation, school-initiated or caregiver-initiated referral, and diagnostic confirmation within primary care pathways. Malaysian studies describe barriers that can delay progression from early concerns to assessment, including variable autism literacy and awareness, variability in parental help-seeking, limited training and confidence among frontline primary care providers, and uneven access to specialist services [18–21]. Importantly, delays may occur at multiple points in the pathway, including delayed caregiver recognition, delayed presentation to services, delayed referral from primary care, and limited capacity for timely confirmatory assessment after referral. The evidence also indicates that delays can persist even after screening, emphasising that screening must be paired with adequate assessment capacity and efficient referral processes to reduce time to diagnosis [30]. Such delays can prolong parental uncertainty and stress and postpone access to interventions that support improved developmental trajectories. Similarly, international evidence similarly shows that many children are diagnosed later than recommended despite early developmental signs [30, 31], with pooled estimates suggesting a mean age at diagnosis of approximately 60 months in studies published between 2012 and 2019 [32].

When screening is implemented early and linked with timely access to evidence-based intervention, children can enter services sooner and achieve better developmental gains across key domains, including social communication, language, cognition, and adaptive functioning [2, 7, 33–37]. Earlier intervention can also strengthen functional and adaptive skills, provide families with timely guidance, and reduce later reliance on more intensive support by promoting greater independence and participation in everyday settings, which is consistent with a rights-based approach to inclusion [20, 38]. In Kelantan, the age pattern observed in this study reinforces the operational priority of strengthening early identification in primary care through consistent screening implementation, improved clinician competency in developmental surveillance, and clear referral mechanisms for children who require further assessment.

Birth order was also independently associated with ASD diagnosis in this study, with first-born children being more likely to receive an ASD diagnosis than later-born children. Similar patterns have been reported in a Malaysian case-control study and in population-based analyses from other settings, although the magnitude and direction of the association vary across populations [28, 39]. Several pathways may explain this pattern. Biologically, first pregnancies differ from subsequent pregnancies and may involve distinct maternal immune and inflammatory profiles and differences in placental function that could influence the intrauterine environment and fetal neurodevelopment [28, 40]. Some studies also suggest that first deliveries may involve greater exposure to intrapartum stressors, including birth-related complications and episodes of reduced oxygenation, which could contribute to subtle neurodevelopmental vulnerability relevant to ASD developmental pathways [41]. However, evidence remains heterogeneous, and an increased likelihood of ASD diagnosis has also been reported among later-born children, particularly in relation to very short or prolonged interpregnancy intervals [42]. From a primary care perspective, birth order may also capture ascertainment processes: first-time parents may monitor milestones more closely and raise concerns more readily, whereas developmental differences among later-born children may be normalised or interpreted in relation to older siblings, potentially influencing help-seeking and the likelihood of referral. These findings reinforce the importance of applying developmental surveillance consistently across all children, including later-born children, to minimise inequities related to variation in parental experience and consultation behavior.

Although ASD is widely reported to be more common among boys, male sex was not significantly associated with ASD diagnosis in this study. Among children newly diagnosed with ASD, the male-to-female ratio was approximately 1.6:1, which is lower than the ratio often reported in population-based ASD studies. This finding may reflect the clinic-based sampling frame, the modest number of ASD cases, and the recruitment of children who were already presenting with developmental concerns or referred for further assessment. Therefore, the absence of a statistically significant sex effect should be interpreted cautiously and does not contradict the established male predominance reported in the broader ASD literature.

From a primary care service perspective, the study findings highlight the need for more systematic developmental surveillance at all routine child health visits, rather than relying primarily on parental complaints or spontaneous help-seeking. Strengthening early identification requires targeted training for medical officers and other frontline healthcare providers to recognise early ASD features, use structured screening approaches appropriately, and communicate concerns effectively with caregivers. In parallel, primary care clinics require clear, time-bound referral pathways to Family Medicine Specialist services and paediatric or developmental teams, supported by feedback mechanisms that confirm referral receipt, track attendance, and ensure that children who screen positive progress to confirmatory assessment. Improving coordination across levels of care may reduce delays in referral and diagnosis and support earlier access to intervention in the Kelantan setting.

This study has several limitations. First, the cross-sectional design precludes causal inference, and the observed associations may reflect service use, recognition, referral, and diagnostic pathways rather than underlying biological mechanisms. Second, the use of purposive sampling from high-volume government primary care clinics may have introduced selection bias, as children attending these facilities may differ from those who do not access government primary care or who seek care through private or specialist services. Therefore, the findings should not be generalised to all children in Kelantan or to the wider child population.

In addition, the sample included a relatively large proportion of older children, with a mean age of 7.38 years and more than one-third aged 10–12 years. This age distribution may reflect delayed recognition, delayed presentation, school-initiated referral, or referral-driven ascertainment within primary care pathways. However, it may also have been influenced by clinic attendance patterns, invitation processes, and caregiver willingness to participate during the study period. In the present study, some parents of younger children declined participation because extending clinic time for study procedures was difficult when children became restless or distressed during clinic visits. These factors should be considered when interpreting the association between older age and ASD diagnosis.

Diagnostic assessment was conducted by trained Family Medicine Specialists based on DSM-5 criteria. However, the involvement of multiple assessors without formal inter-rater reliability assessment may have introduced some variability in diagnostic classification. This should be considered when interpreting the ASD and non-ASD classification used as the outcome in the regression analysis. Given the modest number of ASD cases, the multivariable model was kept parsimonious, and the adjusted estimates should be interpreted cautiously. While the S-BP demonstrated favourable validation findings in the broader study, further external validation in other Malaysian primary care settings is warranted before wider implementation. Nonetheless, the findings provide practical information for strengthening primary care services in Kelantan, where earlier identification will likely depend on systematic surveillance, strengthened clinician capacity, efficient referral processes, and community engagement to support timely help-seeking.

## Conclusion

This study adds local evidence on factors associated with ASD diagnosis among children attending government primary care clinics in Kelantan. The association with older age should be interpreted as a service-pathway finding, suggesting possible delays in recognition, presentation, referral, or diagnostic confirmation, rather than as evidence of etiologic risk. The findings reinforce the need to strengthen how ASD is identified and managed within routine primary care services, particularly in settings where developmental concerns may present across a wide age range.

Moving forward, developmental surveillance in primary care should be implemented more systematically across all child health visits, supported by targeted training for medical officers to recognise early ASD features and apply structured screening appropriately. Clear, time-bound referral pathways with feedback and follow-up mechanisms are also essential to reduce delays between initial concern, referral, and confirmatory assessment. Strengthening these service components can shorten the time to diagnosis and improve the likelihood that children access early intervention at the most beneficial stage of development.

## Data Availability

The data underlying the findings contain sensitive human participant information. Due to ethical and confidentiality restrictions imposed by the Medical Research and Ethics Committee Ministry of Health Malaysia and the data custodian, the dataset is not publicly available. De-identified data may be made available to qualified researchers upon reasonable request and subject to approval by Ministry of Health, Malaysia.
